# ROCK1 as a novel prognostic marker in vulvar cancer

**DOI:** 10.1186/1471-2407-14-822

**Published:** 2014-11-07

**Authors:** Erica M Akagi, André M Lavorato-Rocha, Beatriz de Melo Maia, Iara S Rodrigues, Kátia C Carvalho, Monica M Stiepcich, Glauco Baiocchi, Yukie Sato-Kuwabara, Silvia R Rogatto, Fernando A Soares, Rafael M Rocha

**Affiliations:** Molecular Morphology Laboratory, Investigative Pathology, AC Camargo Cancer Center, São Paulo, SP Brazil; Department of Obstetrics and Gynecology, School of Medicine of São Paulo University, São Paulo, SP Brazil; Pathology Department, Fleury Institute, São Paulo, SP Brazil; Department of Gynecology Oncology, AC Camargo Cancer Center, São Paulo, SP Brazil; Department of Anatomic Pathology, AC Camargo Cancer Center, São Paulo, SP Brazil; São Paulo and Department of Urology, Faculty of Medicine, UNESP, NeoGene Laboratory, AC Camargo Cancer Center, Botucatu, SP Brazil; Molecular Morphology Laboratory, AC Camargo Cancer Center, Rua Antônio Prudente, 109. 1o Andar, Patologia Investigativa, Liberdade, São Paulo, SP CEP: 01509-900 Brazil

**Keywords:** Vulvar carcinoma, ROCK1, qRT-PCR, Immunohistochemistry, aCGH, Prognosis

## Abstract

**Background:**

Vulvar carcinoma is an infrequent tumour, accounting for fewer than 3% of all malignant tumours that affect women, but its incidence is rising in the past few decades. In young women, the manifestation of the vulvar carcinoma is often linked to risk factors such as smoking and HPV infection, but most cases develop in women aged over 50 years through poorly understood genetic mechanisms. Rho-associated coiled-coil-containing protein kinase 1 (ROCK1) has been implicated in many cellular processes, but its function in vulvar cancer has never been examined. In this study, we aimed to determine the prognostic value of ROCK1 gene and protein analysis in vulvar squamous cell carcinoma (VSCC).

**Methods:**

ROCK1 expression levels were measured in 16 vulvar tumour samples and adjacent normal tissue by qRT-PCR. Further, 96 VSCC samples were examined by immunohistochemistry (IHC) to confirm the involvement of ROCK1 in the disease. The molecular and pathological results were correlated with the clinical data of the patients. Sixteen fresh VSCC samples were analyzed by array-based comparative genomic hybridization (aCGH).

**Results:**

In each pair of samples, *ROCK1* levels were higher by qRT-PCR in normal tissue compared with the tumour samples (p = 0.016). By IHC, 100% of invasive front areas of the tumour and 95.8% of central tumour areas were positive for ROCK1. Greater expression of ROCK1 was associated with the absence of lymph node metastasis (p = 0.022) and a lower depth of invasion (p = 0.002). In addition, higher ROCK1 levels correlated with greater recurrence-free survival (p = 0.001). Loss of ROCK1 was independently linked to worse cancer-specific survival (p = 0.0054) by multivariate analysis. This finding was validated by IHC, which demonstrated enhanced protein expression in normal versus tumour tissue (p < 0.001). By aCGH, 42.9% of samples showed a gain in copy number of the ROCK1 gene.

**Conclusions:**

ROCK1 is lower expressed in tumour tissue when compared with adjacent normal vulvar epithelia. In an independent sample set of VSCCs, lower expression levels of ROCK1 correlated with worse survival rates and a poor prognosis. These findings provide important information for the clinical management of vulvar cancer.

## Background

Vulvar carcinoma is an infrequent tumour, accounting for 3% to 5% of all cancers of the female genital system [1–3]. Its incidence rises with age, peaking in women aged between 65 and 75 years [4, 5].

Vulvar squamous cell carcinoma (VSCC) constitutes 90% of all malignant vulvar tumours and has 2 clinicopathological types. The first type arises primarily in younger patients and is associated with human papillomavirus (HPV) infection; the other form is seen mostly in elderly patients and appears to develop independently of HPV infection. These types of VSCC have disparate epidemiological, clinical, pathological, and molecular characteristics [3, 4, 6]. Despite its rarity, the incidence of VSCC has been rising in the past several decades, necessitating the identification of predictive factors of its prognosis.

Changes in cellular dynamics induce morphological alterations in cells, due to reorganization of the actin cytoskeleton. The Rho family of small GTPases are central regulators of the dynamics and reorganization of the actin cytoskeleton, mediating the formation of stress fibers and focal adhesions [7–9]. Certain members of the Rho family, such as RhoA and RhoC, interact with downstream targets, culminating in various cellular responses. Their principal activity is to promote actomyosin contractility by phosphorylating a specific serine/threonine kinase, Rho-kinase associated coiled-coil (ROCK).

ROCK1 and 2 have been implicated in many cellular processes and pathologies, particularly in metastatic processes of cell lines and in the cardiovascular and nervous systems. Based on their oncogenic activity, ROCKs are being examined as therapeutic targets in various tumours, such as non-small-cell lung tumours [10]; glioblastoma [11]; osteosarcoma [12]; and prostate [13, 14], breast [15], ovarian [16], hepatocellular [17], and bladder cancers [18].

Human *ROCK1* maps to chromosome 18 (18q11.1) [19–22] and performs its functions by phosphorylating substrates, such as myosin light chain (MLC), the MLC phosphatase subunit (MYPT-1), and LIM kinase; many other substrates continue to be reported. These substrates catalyze many processes during morphological changes and metastasis, including structural rearrangement, adhesion, alterations in cellular polarity, migration, invasion, transformation, proliferation, cytokinesis, and apoptosis [9, 22, 23].

The precise function of *ROCK1* in carcinogenesis and in the architectural rearrangement of tumour cells during metastasis remains debated [24]. *ROCK1* appears to be implicated in a complex balance between oncogene function and proapoptotic responses, depending on the cell type. Based on its involvement in cell migration in other tumours and the lack of data on its function in vulvar carcinomas, we selected *ROCK1* for further study.

We aimed to examine the function of ROCK1 in the progression of vulvar carcinoma. In this study, we measured ROCK1 mRNA and protein levels and analyzed the data on ROCK1 copy number alterations from a parallel project of our group. The transcript and protein results were correlated with clinicopathological characteristics to determine the prognostic value of ROCK1 in vulvar cancer.

## Methods

### Patient and sample selection

A total of 96 invasive vulvar carcinoma samples were randomly and retrospectively selected from the archives of the AC Camargo Cancer Center Anatomic Pathology Department from January 1990 to December 2010 and analyzed by immunohistochemistry. All samples were formalin-fixed and paraffin-embedded (FFPE), and their HPV status has been reported [2, 5, 25]. Sixteen fresh frozen tumour samples and 11 adjacent nontumour samples were also obtained from the AC Camargo Cancer Center Biobank for mRNA expression and DNA copy number analysis.

The inclusion criteria were patients who had undergone surgery or biopsy in this hospital and were diagnosed with invasive vulvar squamous cell carcinoma. All cases were H&E-stained and reviewed by experienced pathologists to confirm the previous diagnosis and adapt the reports to updated nomenclature. The clinical data on all patients were obtained from their medical records. In situ carcinomas, cases in which neoadjuvant radiotherapy and/or chemotherapy were performed, and cases that lacked sufficient material or clinical information for the analyses were excluded from the study.

This work was approved by the ethics committee at AC Camargo (Research Ethics Committee number 1672/12) and was performed per the Helsinki Declaration.

### RNA extraction from fresh frozen samples

The RNeasy Mini Kit RNA Extraction Kit (QIAGEN, Austin, TX, USA) and a Precellys® 24 homogenizer (Stretton Scientific, Stretton, UK) were used to extract RNA from the fresh frozen samples per the manufacturer’s instructions. Prior to the extraction, the H&E slides from all samples were reviewed by the Biobank’s chief pathologist (Dr. AHJFMC). Aliquots of RNA were stored at -80°C until cDNA synthesis.

### Quantitative real-time RT-PCR (RT-qPCR)

Gene expression was analyzed by RT-qPCR on an Applied Biosystems 7900HT Fast Real-Time PCR System (Applied Biosystems, Foster City, CA, USA) using the TaqMan Universal PCR Master Mix detection system (Applied Biosystems), according to the supplier’s specifications. Primers and probes for *ROCK1* (Hs01127688_m1) were purchased from Applied Biosystems. *HPRT* was used as an endogenous control. Data analyses were performed, comparing adjacent normal and tumorous vulvar samples. The Pfaffl [26] method was used to obtain relative quantification (RQ) values and determine gene expression levels [26].

### Immunohistochemistry

Four-micrometer-thick FFPE samples were placed on StarFrost® electrically charged slides (Braunschweig, Germany). All reactions were performed on whole-tissue slides using the Advance Kit Protocol (DAKO). Antigen recovery was performed using Tris-EDTA (pH 9.0) in a water bath (96°C). The primary antibody was anti-ROCK1 (ABCAM, Cat.#1761-1, Clone EP786Y), diluted 1:100. At the end of the reaction, the slides were washed with tap water, dehydrated sequentially in alcohol and xylene, and mounted manually.

### Evaluation of immunohistochemistry

Slides were digitalized on an APERIO® scanner and scored visually. IHC expression patterns were evaluated quantitatively, wherein expression levels were scored by the percentage of positive cells and the intensity of immunostaining [HScore = Σ (ix Pi) and Pi: percentage of positive pixels, ranging from 0% to 100% and color intensity of the pixel i =0, 1, 2, or 3], ranging between 20 and 250 per Rodrigues et al. [25]. Final HScores were defined as HScore =1 when the positivity was weak, with staining intensity ranging from 20 to 149 and HScore =2 for strong staining and a staining intensity of ≥150. ROCK1 immunostaining was present in all samples.

Two areas for each case—the central tumour and invasive front—were examined for ROCK1 expression. As described (Rodrigues et al. [25]), the central tumour was considered as the largest area of extension of the tumour; at least 3 areas were selected and analyzed. The invasive front was defined as a group of up to 5 cells that detached from the main tumour mass, which usually infiltrated the adjacent stroma; 10 fields were selected [25].

### Array based-comparative genomic hybridization array (aCGH)

Based on our ROCK1/mRNA and protein data, we examined ROCK1 copy number alterations in vulvar carcinoma samples by array-CGH using data from a parallel study. A total of 200 ng each of tumour DNA and normal commercially available DNA (Human Genomic DNA: Female; Promega, Madison, USA) were analyzed compared on an 8 × 60 K Agilent platform for aCGH (Agilent Technologies®, Santa Clara, USA).

The labeling, hybridization, and washes were performed per the Agilent Oligonucleotide Array-Based CGH for Genomic DNA Analysis – Enzymatic Labeling kit protocol (Agilent Technologies®, Santa Clara, USA). The slides were scanned on a DNA microarray scanner with Surescan High-Resolution Technology (Agilent Technologies®, Santa Clara, USA), based on HG19, and the results were extracted using Feature Extraction, v10.7.3.1 (Agilent Technologies®, Santa Clara, USA). Copy number analysis was performed using Nexus Copy Number Software, v6.0 (Biodiscovery, El Segundo, USA).

A copy number alteration was defined as exceeding the significance threshold of 1 × 10^−6^ in a minimum of 5 consecutive probes and in more than 30% of the samples.

Thresholds were defined as the average log_2_ CGH fluorescence ratio for copy gains ≥0.3, high copy number gains defined as ≥0.6, losses defined as ≤ -0.3, and homozygous losses defined as ≤ -1.0. Nonrandom genomic copy number alterations were identified using the Fast Adaptive States Segmentation Technique 2 (FASST2) algorithm and the Significance Testing for Aberrant Copy number (STAC) statistical method [27, 28]. Alterations that were detected in at least 42.9% of samples were examined in greater detail.

### Statistical analysis

Statistical analyses were performed using the Statistical Package for Social Sciences (SPSS, IBM), version 20.0. Protein expression in the tumour center and invasive front was compared by Wilcoxon signed-rank test. Mann-Whitney test and student t-test were used to analyze the association between protein expression and clinicopathological parameters, and the Kaplan-Meier method was used to examine specific cancer survival and recurrence-free survival rates. The difference between survival curves was assessed by log-rank test. Multivariate analyses were performed using the Cox proportional hazards regression model. Statistical significance was set to p ≤0.05.

## Results

### Demographic and clinicopathological features

The mean age of the 96 patients was 75 years, ranging from 30 to 103 years. The mean age at menopause was 50 years, ranging between 38 and 60 years. Most patients were Caucasian (83.3%) and did not consume alcohol (88.5%); 15.6% of patients were current or past smokers.

Forty eight percent of the patients were HPV-positive, most of whom had the subtypes HPV16 (48%), HPV33 (24%), and HPV18 (15%). Associated lesions were present, such as vulvar intraepithelial neoplasias (VINs; 13.5% of patients) and lichen sclerosus (6.25%). Based on the histological diagnosis, moderately differentiated squamous cell carcinomas (SCC2) was the most prevalent form (46%), followed by SCC1 (34%), basaloid (9%), SCC3 (8%), sarcomatoid (2%), and verrucous carcinoma (1%). Most tumours were classified as FIGO stage IB (53.1%), followed by stage IIIB (20.3%), IIIA (12.1%), II (6.2%), IIIC (6.2%), and IVA (2.1%). Of the 96 patients with VSCC, 34.4% died due to the cancer, and 46.9% expired due to other causes.

### ROCK1 mRNA expression

**Figure 1:**
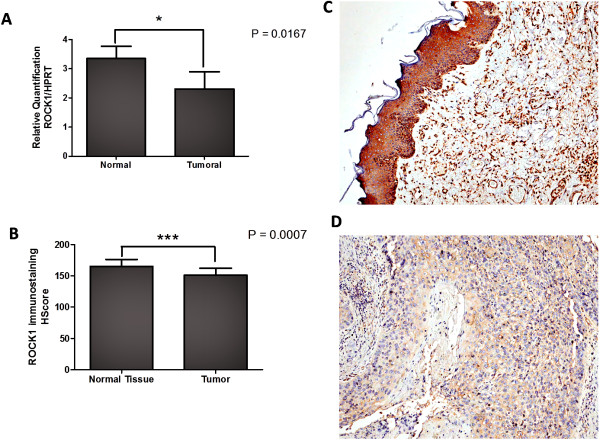
**ROCK 1 mRNA and protein are overexpressed in normal tissues.** ROCK1 is overexpressed by qRT-PCR analysis in normal adjacent samples compared with tumour samples (p =0.016, **A**). Increased ROCK1 immunostaining HScore in normal epithelium compared with tumour in a subset of samples (n = 21) **(B)**; p <0.001. Representative images of normal epithelium **(C)** and tumour **(D)** immunostaining from the same case, captured at 400× magnification.

By RT-qPCR, *ROCK1* was overexpressed in normal adjacent samples compared with the tumour tissue (p = 0.0167, Figure 1A). Also, ROCK1 HScores were higher in normal epithelium versus the tumour areas in a subset of samples (n = 21) (p < 0.001) (Figure 1B, C, and D).

### ROCK1 immunostaining

**Figure 2:**
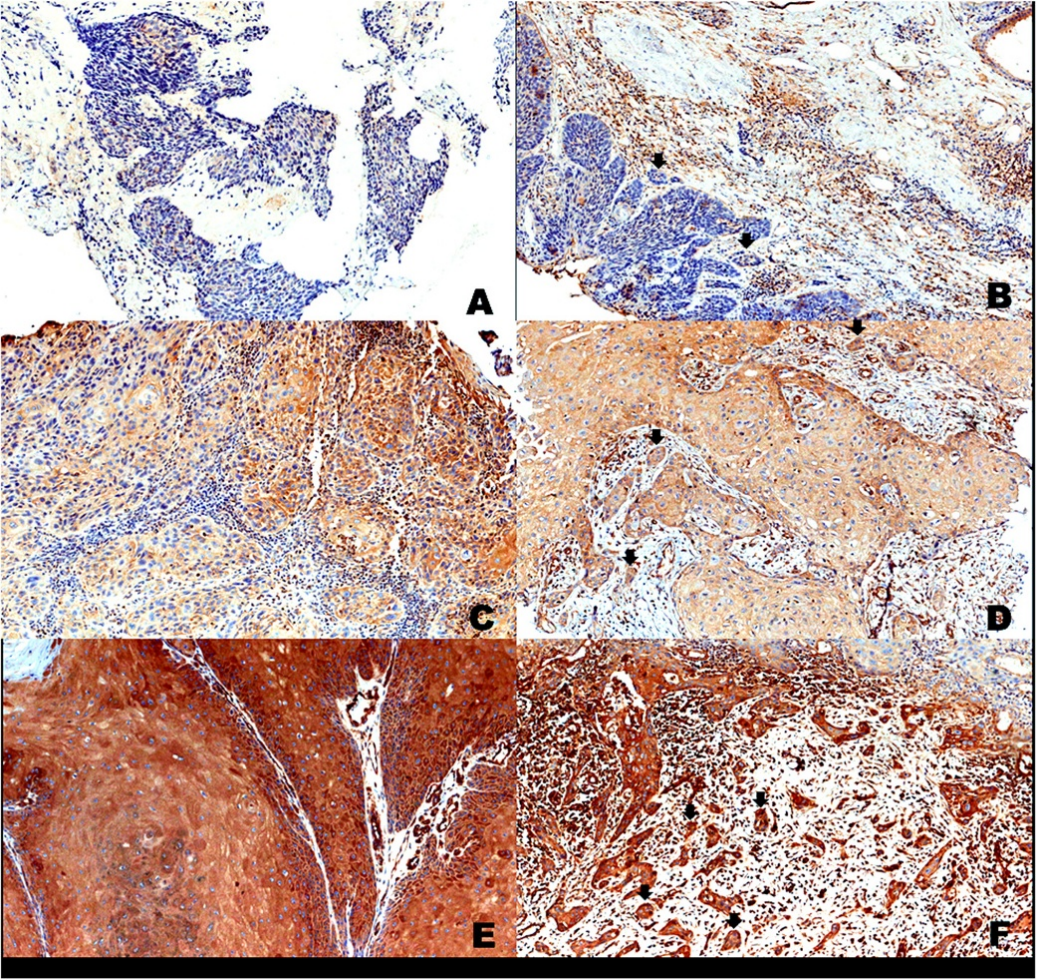
**Immunohistochemical staining of ROCK1 in vulva carcinoma.** Representative image of weak-positive staining in central tumour **(A)** and invasive front (**B**, arrows); moderate staining in the central tumour **(C)** and invasive front (**D**, arrows); strong-positive staining in central magnification. Images **A** and **B** were captured at 200× magnification. Images **C**, **D**, **E**, and **F** were captured at 400× magnification.

**Figure 3:**
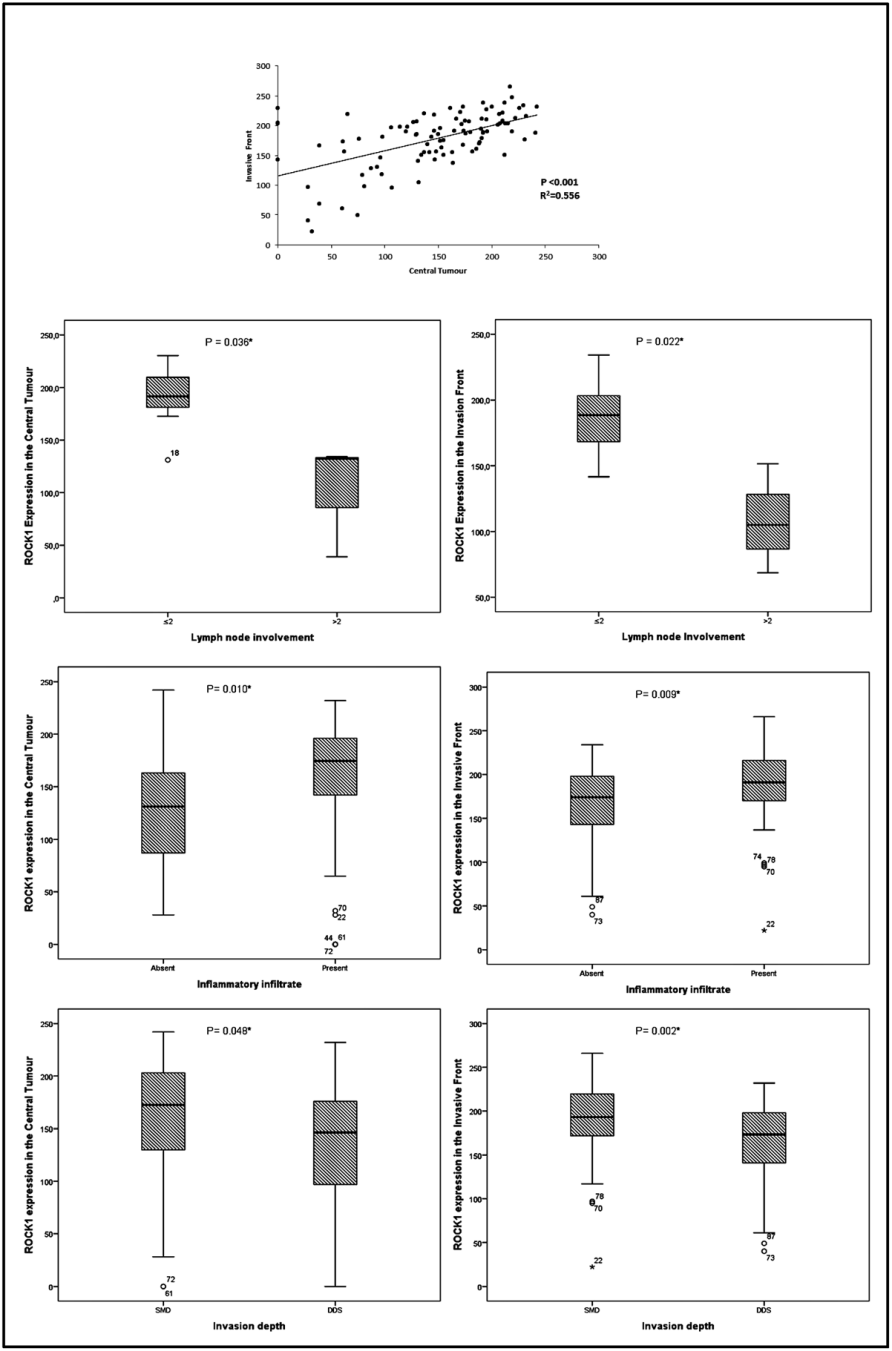
**Association between clinicopathological features and ROCK1 in vulvar carcinoma.** Abbreviations: ≤2 = 2 or fewer lymph nodes involved; >2 = more than 2 lymph nodes involved; SMD = superficial and mid-dermis; DDA = deep dermis and subcutaneous tissues. *Statistically significant, p < 0.05.

ROCK1 immunostaining was heterogeneous and cytoplasmic in all tumour extensions (Figure 2) and positive in the invasive front of all cases (100%) and in 92 central tumours (95.8%). There was a significant positive correlation between central tumour and invasive front expression of ROCK1 (p <0.001; Figure 3A).

In the statistical analysis, greater expression of ROCK1 in central tumours and the invasive front correlated significantly with the absence of lymph node metastasis (p = 0.036 and p = 0.022, respectively), the presence of inflammatory infiltrate (p =0.010 and p = 0.009, respectively), and a lower depth of invasion (p = 0.048 and p = 0.002), as shown in Figure 3B. There was no association between ROCK1 positivity and HPV infection, histological type, FIGO stage, recurrence, or vascular invasion.

### ROCK1 expression and patient survival

**Figure 4:**
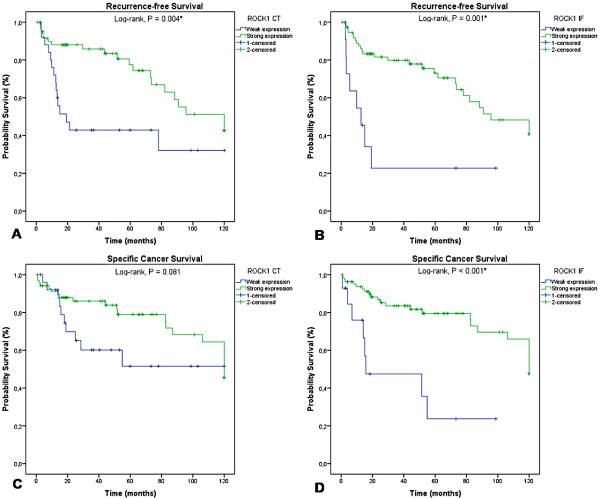
**Kaplan-** **Meier survival curves for ROCK1 staining.** Increased expression of ROCK1 in the central tumour (p =0.004; **A**) and in the invasive front (p =0.001; **B**) correlates with better recurrence-free survival and lower cancer-specific survival in the central tumour (p =0.081; **C**) and in the invasive front (p <0.001; **D**). Abbreviations: CT = central tumour; IF = invasive front.

Patients with lower expression of ROCK1 in the central tumour and invasive front had lower recurrence-free survival rates (p = 0.004 and p = 0.001, respectively; Figure 4A and B), and those with weak ROCK1 expression in the invasive front experienced lower cancer-specific survival (p <0.001; Figure 4C and D). By multivariate analysis, high ROCK1 expression in the invasive front was independently associated with greater cancer-specific survival (HR 0.3, 95% CI 0.11–0.84, p = 0.0054; Table 1). The clinicopathological characteristics were analyzed by Cox regression. Lymph node metastasis (data not shown) and vascular invasion were independently associated with decreased of the survival (p = 0.0074 and 0.0365, respectively).

**Table 1:** **Multivariate analysis of ROCK1 expression and clinicopathological characteristics in patients with vulvar SCC**

Variables	Category	n	Hazard ratio for survival	95.0% CI	p
Histologic types	SCC1, SCC2, Verrucous Ca (1)	76	1	1	0.6177
	SCC3, Basaloid Ca, Sarcomatoid Ca(2)	20	0.78	0.30 - 2.05	
FIGO stage	IA, IB, II (1)	54	1	1	0.2083
	IIA, IIIB, IIIC, IVA, IVB (2)	40	1.62	0.76 - 3.47	
HPV	Absent (0)	50	1	1	0.2238
	Present (1)	46	0.63	0.30 - 1.33	
Inflammatory infiltrate	Absent (0)	38	1	1	0.4964
	Present (1)	58	0.77	0.36 - 1.64	
Vascular Invasion	Absent (0)	74	1	1	0.0365*
	Present (1)	17	2.22	1.03 - 4.77	
Perineural invasion	Absent (0)	76	1	1	0.4178
	Present (1)	12	1.43	0.60 - 3.38	
ROCK1 CT	Weak expression	22	1	1	0.6653
	Moderate expression	55	0.70	0.28 - 1.75	
	Strong expression	19	0.95	0.34 - 2.67	
ROCK1 IF	Weak expression	8	1	1	0.0054*
	Moderate expression	53	**0.22**	0.08 - 0.60	
	Strong expression	35	**0.30**	0.11 -0.84	

*Abbreviations*: *SCC* squamous cell carcinomas, *CI* confidence interval, *FIGO* International Federation of Gynecology and Obstetrics, *HPV* human papillomavirus, *SMD* superficial and mid dermis, *DDA* deep dermis and subcutaneous tissues, *CT* central tumour and *IF* invasive front. *Statistically significant, p < 0.05.

### aCGH analysis

**Figure 5:**
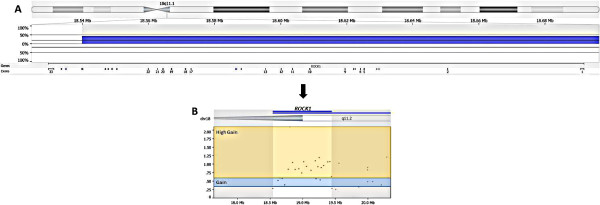
**Representative image of aCGH analysis of chromosome 18 with emphasis on** ***ROCK1*** **.** **(A)** The copy number gain (chr18:18,539,853-19,429,001) is in blue; exons of *ROCK1* are illustrated at the bottom of the diagram. **(B)** Example highlighting the gains (≥0.3) in blue and high gains (≥0.6) in yellow. Gene regions covered by each probe can be seen as small dots.

By aCGH analysis, 29 regions underwent significant copy number alterations: 9 were associated with copy number loss (8p23.3, 5q11.1-q11.2, 3p11.1-q11.1, 9p23, 21p11.2-p11.1, Xq28, 7q36.3, 19p13.3, and 21p11.2), and 20 had gains (1q22, 20q11.21-q11.23, 1p36.23-p36.22, 11q13.3, 19q13.12, 19q13.32, 7q11.21, 7q11.22, 7q11.23, 7q11.23, 7q22.1, 11q12.2-q12.3, 11q12.3, 11q13.2, 16q22.1, 18q11.1-q11.2, 18q11.2, 7p22.2-p22.1, 12q24.31, and 15q11.1-q11.2). Of the latter, region 18q11.1-q11.2, which harbors *ROCK1*, had more copies than the reference DNA in 42.9% of samples (Figure 5).

To identify their function and the processes that they mediate, the genes that were selected in the copy number analysis were included in an *in silico* functional analysis, performed by Ingenuity Pathways Analysis (IPA). ROCK1 appeared in 2 of the top 5 canonical pathways with the highest ratios—RhoGDI and Rho GTP signaling—both of which are associated with cell migration.

## Discussion

ROCK has significant functions in cancer progression and metastasis, rendering it a potential therapeutic target [9]. In this study, we examined the function of the Rho-associated protein kinase ROCK1 in human vulvar carcinoma. Our data showed that aspects that are related to a good prognosis, such as the absence of lymph node metastasis, lower depth of invasion, and better survival, correlated with ROCK1 immunoexpression, suggesting that ROCK1 is a marker of good prognosis in vulvar cancer.

ROCK1 immunopositivity was observed in the tumour invasion fronts of all cases and in nearly all central tumour areas. Also, there was no difference in ROCK1 expression levels between central tumour versus invasive front areas, in contrast to what we have reported concerning the variability of epithelial to mesenchymal transition markers [26], EGFR [29], and c-Kit [5] in tumours. Nevertheless, vulvar carcinomas can be highly heterogeneous [29, 30], and cytoplasmic immunostaining for ROCK1 protein was heterogeneous in all tumour extensions in our cases, reflecting a disadvantage of ROCK-targeting therapies in this tumour type.

In this study, we performed a global evaluation of ROCK1 expression and its relationship with clinical data and the prognosis. ROCK has a significant influence on cancer progression [8, 9, 11, 31], metastasis [17, 21, 32, 33], and apoptosis [12, 23, 34]. Recent evidence suggests that ROCK phosphorylates PTEN [34, 35], a negative regulator of the PI3-K/Akt pathway, with roles in cell survival and apoptosis [34, 36].

Inhibition of ROCK/Rho-kinase in Ras-transformed cells is insufficient to effect a motile phenotype in them, suggesting that this cell type requires changes in other regulators of the cytoskeleton to increase its motility [32]. Notably, elongated cells, such as SW-962, a vulvar squamous cell carcinoma metastatic cell line do not require Rho or ROCK function, unlike cells that move through rounded, or amoeboid, movement [21, 37, 38].

Thus, we hypothesize that elongated vulvar carcinoma cells move and migrate using mechanisms other than Rho/ROCK activation. The effectiveness of therapeutic agents against ROCK, such as fusadil and Y27632, might be limited when cells move through elongated morphology [37].

ROCK1 immunostaining was also associated with important clinical features in vulvar cancer and with the most significant clinical property and prognostic factor in this tumour: lymph node metastasis. When overexpressed, ROCK1 correlated inversely with lymph node metastasis in the central tumour and invasive front. To define groups for the statistical analysis regarding the clinical implications of the presence or absence of lymph node involvement, we considered positivity as metastasis when 2 or more lymph nodes were involved and negativity when 1 or 0 lymph nodes were involved. This strategy was based on a previous study that demonstrated that 5-year survival for patients with negative or 1 positive lymph node did not differ from each other [39]. Similarly, greater expression in the central tumour and invasive front was associated with lower invasive depth and higher recurrence-free survival.

Clinicopathological characteristics analyzed by Cox regression demonstrated that lymph node metastasis and vascular invasion were independently associated with decreased of the survival, indicating that these features are related to poor survival in patients with VSCC. Also, higher expression of ROCK1 was linked to greater survival, the absence of lymph node metastasis, and a lower depth of invasion. Moreover, elevated ROCK1 levels in the invasive front was an independent protective factor (HR =0.22 for moderate expression, and HR =0.33 for strong expression) with regard to cancer-specific survival (p =0.0054).

These results implicate ROCK1 as a good prognostic marker in vulvar cancer. In addition, patients with weak expression of ROCK1 in the invasive front, but not the central tumour, had lower cancer-specific survival rates, implying that this marker is protective during cancer progression. Conversely, ROCK1 has been largely reported as a marker of worse prognosis in many cancer types [12].

There is emerging evidence that ROCK governs the morphological events that take place during apoptosis (cell contraction, membrane blebbing, nuclear fragmentation, and disintegration of apoptotic cells) through cytoskeletal rearrangement and actomyosin contractility [40, 41]. Other groups contend that ROCK1 is required for apoptotic fragmentation and phagocytosis of dying COS-7 cells [41]. Because ROCK is a proapoptotic regulator in various cell types, depending on the cell type and apoptotic stimulus [40], we believe that its overexpression in vulvar cancer is associated with apoptotic stimuli and, thus, it can be associated with better prognosis; as demonstrated in other studies [41, 42]. Although the relationship between apoptosis and the prognosis remains unknown, it could, at least in part, explain the association of ROCK1 with a good prognosis in vulvar cancer.

ROCK1 copy number gains were detected in 42.9% of our samples. However, these data are controversial, because ROCK1 was more highly expressed in normal tissue by IHC and RT-qPCR compared with tumour samples. Although associations between copy number and gene expression comprise the concomitant amplification of the gene with enhancement of its expression, there remain other genes, approximately 50% [43], the amplification of which does not correspond to gene overexpression.

We hypothesize that tissues that were used for normalization of the validation techniques were adjacent to the tumour. Despite careful morphological analysis by an experienced pathologist of nontumour tissue, the proximity of malignant cells could have influenced the surrounding microenvironment, upregulating various genes, including ROCK1, in normal epithelia.

In addition, other mechanisms, such as microRNA regulation and epigenetic alterations (including methylation and histone deacetylation), might cause the lack of correlation between the genomic and proteomic data found in our study. Previous reports have demonstrated the function of microRNAs (eg, microRNA-135a and microRNA124-3p) in *ROCK1* regulation in prostate, gastric, and bladder carcinoma [44–46].

Also, despite the increase in copy number in tumour samples by aCGH analysis, we can not make any conclusions regarding gene integrity and the extent of its abnormal. Our results do not allow us to conclude much concerning the amplification of ROCK1 in the tumour samples or on the possible generation of aberrant mRNA or truncated protein. The genomic mechanism that leads to gene amplification in tumour cells remains undefined, as do the molecular pathways that effect amplified gene expression.

## Conclusions

This is the first report to demonstrate that ROCK1 correlates with a good prognosis in cancer. Although vulvar carcinomas are rare, this type of cancer can serve as a valuable model in the study of molecular alterations that can be transposed to other types of epithelial neoplasms. Further, novel biomarkers, such as ROCK1, are significant, because its evaluation by IHC in routine practice can help better establish prognosis and select more conservative surgical approaches for this mutilating disease.

## Abbreviations

ROCK1: Rho-associated coiled-coil containing protein kinase 1
VSCC (or SCC): vulvar squamous cell carcinoma
HPV: human papillomavirus
IHC: immunohistochemistry
qRT-PCR: quantitative real-time polymerase chain reaction
aCGH: array-based comparative genomic hybridization
MLC: myosin light chain
FIGO: International Federation of Gynecology and Obstetrics
FFPE: formalin-fixed paraffin-embedded
PTEN: phosphatase and tensin homolog deleted from chromosome ten.
